# Diagnosis of smear-negative pulmonary tuberculosis based on clinical signs in the Republic of Congo

**DOI:** 10.1186/s13104-015-1774-8

**Published:** 2015-12-18

**Authors:** Laure Stella Ghoma Linguissi, Christevy Jeannhey Vouvoungui, Pierre Poulain, Gaston Bango Essassa, Sylvie Kwedi, Francine Ntoumi

**Affiliations:** Fondation Congolaise pour la Recherche Médicale, Cité OMS, villa D6, Djoué, Brazzaville, Republic of Congo; Centre de Recherche Biomoleculaire Pietro Annigoni (CERBA), Labiogene, Université de Ouagadougou, 01 BP 364 Ouaga 01, Burkina Faso; Institut National de la Santé et de la Recherche Médicale U 1134, Paris, France; UMR_S 1134, DSIMB, Université Paris Diderot, Sorbonne Paris Cite, Paris, France; Institut National de la Transfusion Sanguine, DSIMB, Paris, France; UMR_S 1134, Laboratory of Excellence GR-Ex, DSIMB, Paris, France; Centre Antituberculeux de Brazzaville, Programme de Lutte contre la Tuberculose, Brazzaville, Republic of Congo; Capacity for Leadership Excellence and Research, CLEAR, INC, Yaoundé, Cameroon; Faculty of Sciences and Techniques, University Marien Ngouabi, BP 2672 Brazzaville, Republic of Congo; Institute for Tropical Medicine, University of Tübingen, Tübingen, Germany; Faculty of Médecine and Biomedical Sciences, University of Yaoundé I, Yaounde, Cameroon

**Keywords:** Clinical signs, Smear-negative pulmonary tuberculosis, Chest radiography, *Mycobacterium tuberculosis*

## Abstract

**Background:**

The diagnosis of pulmonary tuberculosis (PTB) and smear-negative pulmonary tuberculosis (SNPT) in resource-limited countries is often solely based on clinical signs, chest X-ray radiography and sputum smear microscopy. We investigated currently used methods for the routine diagnosis of SNPT in the Republic of Congo (RoC) among TB suspected patients. The specific case of HIV positive patients was also studied.

**Methods:**

A cross-sectional study was conducted at the anti-tuberculosis center (CAT) of Brazzaville, RoC. Tuberculosis suspects were examined for physical signs of TB. Clinical signs, results from sputum smear microscopy, tuberculin skin test (TST) and chest X-ray were recorded.

**Results:**

Of the 772 enrolled participants, 372 were diagnosed PTB. Cough was a common symptom for PTB and no PTB patients. Pale skin, positive TST, weight loss and chest X-ray with abnormalities compatible with PTB (PTB-CXR) were significant indicators of PTB. Thirty-six percent of PTB patients were diagnosed SNPT. This category of patients presented less persistent cough and less PTB-CXR. Anorexia and asthenia were significant indicators of SNPT. In the case of HIV+ patients, 57 % were SNPT with anorexia, asthenia and shorter cough being strong indicators of SNPT.

**Conclusion:**

Chest X-ray abnormalities, weight loss, pale skin and positive TST were significant indicators of PTB. Anorexia and asthenia showed good diagnostic performance for SNPT, which deserve to be recommended as index indicators of SNPT diagnosis. Duration of cough is also a relevant indicator, especially for HIV+ patients.

## Background

Tuberculosis (TB), an airborne infectious disease, is the second leading cause of death from an infectious disease worldwide, after the human immunodeficiency virus (HIV). In 2013, the TB disease burden estimated by World Health Organization (WHO) was 9 million people and 1.5 million deaths were estimated to be caused by TB [1].

In resource-constrained settings where sputum culture and nucleic acid amplification techniques are not routinely available, diagnosis of pulmonary tuberculosis (PTB) is based on physical examination, clinical signs, sputum smear microscopy and chest X-ray radiography. Sputum smear microscopy is commonly used for diagnosing PTB [2]. It relies on the detection of acid-fast bacilli (AFB) by the Ziehl-Neelsen light microscopy method [3]. This rapid and cheap technique is known for having very good specificity but moderate sensitivity [4]. It is estimated that 42 % of PTB cases are smear-negative pulmonary tuberculosis (SNPT). Although patients with SNPT are less infectious than patients with smear-positive pulmonary tuberculosis (SPPT) [5], they do contribute to PTB transmission and are thought to be responsible for approximately 20 % of TB transmission [6]. In addition, A good number of TB infections in Africa are reported as SNPT [7]. During clinical examination of patients with PTB, the presence of respiratory symptoms [8] and general characteristic signs of TB are assessed [9]. In countries with limited health infrastructure, the diagnosis of PTB, and especially SNPT, is a challenge [10, 11] and relies on the use of clinical algorithms. However, the sensitivity and specificity of these algorithms vary depending on the clinicians experience [12].

TB is also an opportunistic infection for HIV infected patients [13, 14]. The late diagnoses of HIV as well as TB are significant factors of high mortality in TB patients [15]. There is a scarcity of data on SNPT for TB and HIV infected individuals in the Central African region [16].

The Republic of Congo (RoC) is considered as a “high burden” country for TB and HIV infections [17]. In 2013, the incidence of TB was 382 per 100,000 population and 10,776 cases of TB were reported (from which 73 % of cases were PTB) [18]. The same year, 69,000 HIV infected people and 5400 deaths were reported respectively [19]. We recently showed that TB is the leading cause of death among HIV infected patients in Brazzaville and that 70 % of HIV positive TB suspected patients were probably SNPT [4].

Since 2009, the Central African Network on Tuberculosis HIV/AIDS and Malaria (CANTAM) has conducted a number of baseline epidemiology studies with the aim of preparing sites for the conduct on clinical trials. This network is thus generating useful data that can be used to inform policy changes and health systems improvements in the sub-region. The National TB Control Program (NTCP) in the RoC is plagued by lack of resources in terms of prevention, diagnosis and treatment of TB. In order to build a case for the need of new tools and interventions for TB control, it is important to understand the current situation. This study thus aimed at evaluating current and routine methods in PTB and SNPT diagnosis, which, according to the NTCP guidelines, is mostly based on clinical signs and chest X-ray radiography. In addition, we addressed the diagnosis of SNPT in HIV infected patients [10] since delayed diagnosis in this particular population may be an important cause of mortality and morbidity.

## Methods

### Patient selection

A cross-sectional study with prospective enrollment was conducted at the anti-tuberculosis center (CAT) of Brazzaville, RoC, from February to June 2011. Study participants were male and female patients aged from 18 to 70-years-old, who gave a written informed consent to participate in the study. Ethical authorization was obtained in August 2010 (No. 00000067/DGRST/CERSSA) from the institutional ethics committee *Comité d’Ethique pour la Recherche en Science de la Santé* (CERSSA).

### Specimen collection

Demographic data (age, sex, place of residence and occupation) and clinical data were recorded for all participants during a medical interview and a physical examination by a trained clinician of the CAT. Chest radiography and sputum collection were performed according to the national algorithm against TB [20]. Participants provided three sputum specimens over the course of 2 days. The first specimen was collected the day the patient arrived at the CAT (day 1), the second was collected by the patient himself in the early morning of day 2 and the third was collected at the CAT when the patient brought back the early morning specimen (day 2).

### National algorithm against tuberculosis

The national guideline against TB (*Guide national de lutte contre la tuberculose*) [20] in RoC includes screening for common clinical signs and symptoms such as current cough (if self-reported, cough >2 weeks), fever, asthenia (fatigue), anorexia (loss of appetite) and pale skin. A tuberculin skin test (TST) and a X-ray chest radiography were also performed systematically [4]. Sputum samples were tested with the direct Ziehl-Neelsen light and fluorescence microscopy methods and assessed by a trained technician. Abnormal chest X-ray compatible with PTB (termed PTB-CXR) was followed up with HIV testing to all consenting individuals.

### Case definitions

#### Smear-positive pulmonary tuberculosis (SPPT)

Patients with at least two positive sputum samples and clinical signs compatible with PTB were diagnosed as SPPT. These patients were given the standard anti-TB treatment according to the national guideline against TB.

#### Smear-negative pulmonary tuberculosis (SNPT)

Patients with at least two sputum samples negative for AFB were treated with a full course of non-specific chemotherapy for 15 days. After this period, if there was no improvement of patient condition, new sputum samples were collected. If the second sputum analysis was still negative for AFB, a PTB-CXR was noted and clinical signs were compatible with PTB, the patients were diagnosed as SNPT and they were given the standard anti-tuberculosis treatment according to the national guideline against TB. The diagnosing clinician’s experience plays an important role in the diagnosis of SNPT.

### Statistical analysis

Quantitative variables were expressed as median with interquartile range (IQR). Differences among variables were analyzed with the χ^2^ or Fisher’s exact test for categorical variables. Analysis of data was performed using the STATA software package (version 11, StataCorp, Texas, USA). For all potential predictors, unadjusted odd ratios (ORs) were calculated and provided along with their 95 % confidence intervals (95 % CIs). Multivariate analyses were performed to investigate any association between clinical factors and disease. A *p* value <0.05 was considered to be statistically significant.

## Results

### Study population

**Fig. 1:**
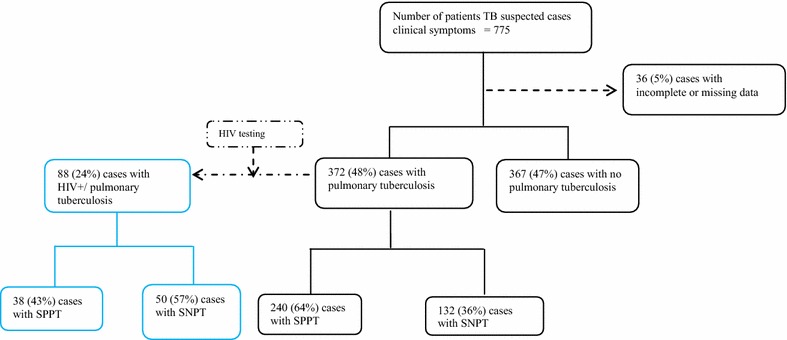
Case distribution flowchart of patients recruited at the *Centre Anti*-*Tuberculeux* in Brazzaville, Republic of Congo

Among the 775 patients enrolled in this study (Fig. 1), 372 (48 %) and 367 (47 %) were diagnosed with PTB and with no PTB respectively. Thirty-six out of the 775 patients (5 %) were excluded because of missing data. None of the recruited patients received previous TB treatment. Two hundred and twenty PTB patients (60 %) were male and the median age was 32-years-old (Table 1).

**Table 1:** Potential predictors for pulmonary tuberculosis in TB suspect individuals

Characteristics	PTB, N = 372	no PTB, N = 367	Unadjusted OR (95 % CI)	LR	R^2^	P value
Men (%)	220 (59)	191 (52)	1.33 (0.99–1.77)	3.77	0.004	0.052
Median age (years) (IQR)	32 (24–42)	34 (25–46)	0.98 (0.97–0.99)	4.68	0.031	0.032
Cough (%)	372 (100)	359 (98)	NA	NA	NA	0.004
Fever (%)	338 (91)	321 (88)	1.42 (0.89–2.28)	2.21	0.002	0.139
Anorexia (%)	299 (80)	298 (81)	0.95 (0.66–1.37)	0.08	0.0001	0.777
Asthenia (%)	334 (90)	314 (86)	1.48 (0.95–2.31)	3.07	0.003	0.082
Pale skin (%)	53 (14)	20 (5)	2.88 (1.69–4.93)	16.62	0.016	<0.001
Weight loss (%)	360 (97)	308 (84)	5.75 (3.03–10.89)	37.93	0.037	<0.001
Positive TST (%)	283 (76)	235 (64)	1.79 (1.30–2.46)	12.84	0.013	<0.001
PTB-CXR (%)	346 (93)	0 (0)	NA	NA	NA	<0.001

*N* number of TB suspects in the group, *NA* not applicable

### Diagnosis of PTB and SNPT

In addition to demographical data, clinical signs (cough, fever, anorexia, asthenia, pale skin, weight loss) and results of TST and CXR examinations were evaluated in their suitability in PTB and SNPT diagnosis.

Cough was common to all PTB cases and to 98 % of the no PTB cases, as would be expected, since this symptom is the major reason for consultation at the CAT. Fever, anorexia and asthenia were not indicators of PTB. However, pale skin, positive TST and weight loss were significant indicators of PTB with OR of 2.88, 1.79 and 5.75 respectively (Table 1). Multivariate analysis showed that patients with pale skin, positive TST and weight loss were 2.76 times more likely to be SNPT cases (OR of 2.76; 95 % CI 1.57–4.86; R^2^ = 0.504). PTB-CXR was found in 93 % of the PTB patients and never in the no PTB patients. PTB patients were split into SPPT and SNPT groups based on the sputum smear assay (Table 2). Out of the 372 PTB cases, 132 (36 %) were diagnosed SNPT after clinical, radiology and sputum smear examinations. SNPT cases had less persistent cough that lasted more than 14 days (OR 0.39; 95 % CI 0.19–0.82) and presented less PTB-CXR (0.18; 95 % CI 0.07–0.44).

**Table 2:** Potential predictors for SNPT in PTB patients

Characteristics	SPPT, N = 240	SNPT, N = 132	Unadjusted OR (95 % CI)	LR	R^2^	P value
Duration of cough above 14 days (%)	226 (94)	114 (86)	0.39 (0.19–0.82)	6.28	0.013	0.012
Median of duration of cough (days) (IQR)	30 (29–61)	30 (21–60)	0.99 (0.98–1.00)	3.37	0.007	0.080
Fever (%)	215 (90)	123 (93)	1.59 (0.72–3.51)	1.38	0.003	0.253
Anorexia (%)	181 (75)	118 (89)	2.75 (1.47–5.14)	11.40	0.024	0.002
Asthenia (%)	209 (87)	125 (95)	2.65 (1.13–6.19)	5.92	0.015	0.025
Pale skin (%)	32 (13)	21 (16)	1.23 (0.68–2.23)	0.46	0.001	0.497
Weight loss (%)	233 (97)	127 (96)	0.76 (0.24–2.45)	0.20	0.0004	0.650
Positive TST (%)	183 (76)	100 (76)	0.97 (0.59–1.60)	0.01	0.000	0.915
PTB-CXR (%)	233 (97)	113 (86)	0.18 (0.07–0.44)	16.44	0.034	<0.01

*N* number of patients in the group

On the opposite, patients with anorexia and asthenia had significantly elevated risk for SNPT with OR of 2.75; 95 % CI 1.47–5.14 and 2.65; 95 % CI 1.13–6.19 (Table 2). Multivariate analysis showed that patients with anorexia, asthenia and a shorter cough were 2.37 times more likely to be diagnosed SNPT (OR of 2.37; 95 % CI 1.15–4.87; R^2^ = 0.355).

### Diagnosis of SNPT for HIV positive patients

**Fig. 2:**
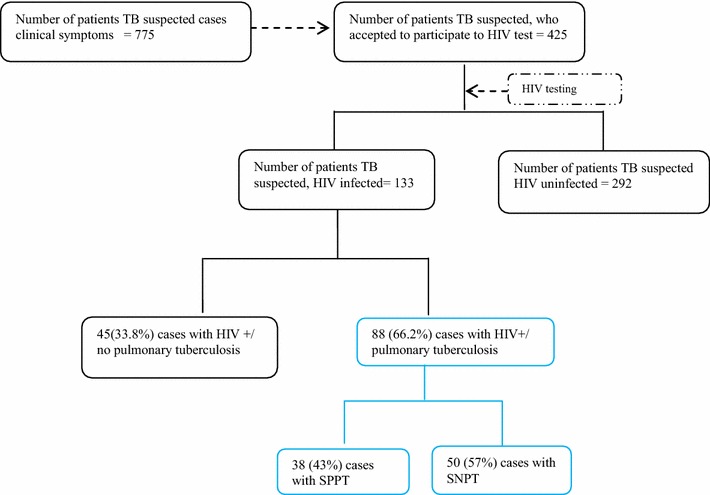
Case distribution flowchart of HIV infected patients recruited at the Centre Anti-Tuberculeux in Brazzaville, Republic of Congo

**Fig. 3:**
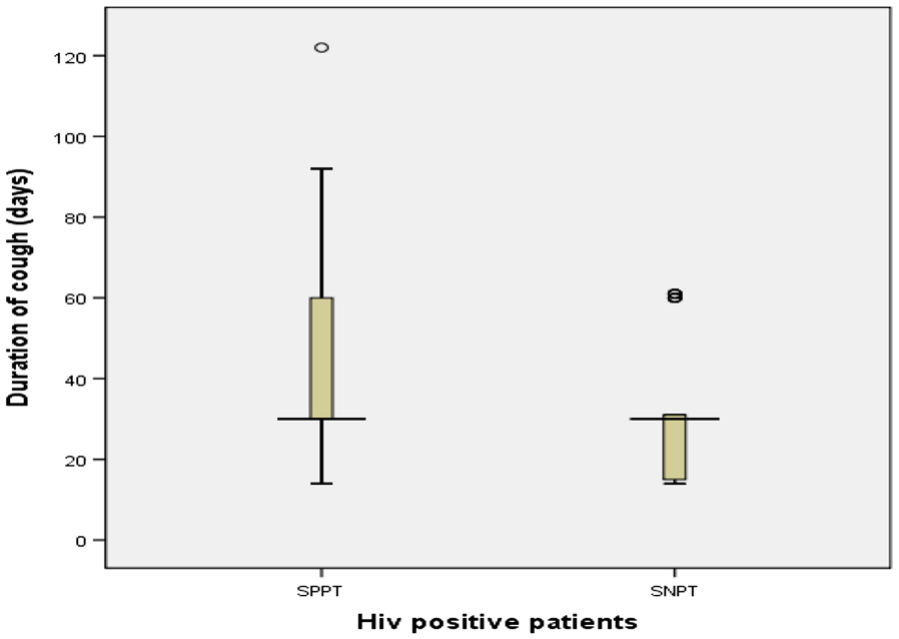
Distribution of the cough duration for SPPT and SNPT in HIV infected patients. *Circles* are outliers excluded from the calculation of the median and IQR

Eighty-eight (24 %) PTB patients presented a positive HIV serology (Fig. 2) and were investigated in more details. Performances of putative indicators regarding the prediction of SNPT cases are presented in Table 3. Fifty-seven percent of PTB/HIV+ patients were diagnosed as SNPT. The median duration of cough was the same for both SPPT and SNPT groups (30 days). However, the IQR was opposed as shown by the distribution of duration of cough in Fig. 3. SPPT patients had longer duration of cough as compared to SNPT cases. SNPT/HIV+ patients were eight times more likely to have anorexia and nine times more likely to have asthenia (OR 8.57; 95 % CI 1.75–41.9 and OR 9.19; 95 % CI 1.06–79.93). In addition, SNPT/HIV+ cases were 20 times less likely to present an abnormal chest X-ray compatible with PTB.

**Table 3:** Potential predictors for pulmonary tuberculosis in HIV infected patients

Characteristics	SPPT, N = 38	SNPT, N = 50	Unadjusted OR (95 % CI)	LR	R^2^	P value
Men (%)	19 (50)	19 (38)	0.61 (0.26–1.44)	1.27	0.011	0.262
Median age, years (IQR)	37.5 (29–44)	39 (29–44)	1.02 (0.98–1.07)	1.11	0.0093	0.296
Median duration of cough (days) (IQR)	30 (30–60)	30 (15–31)	0.99 (0.97–1.01)	1.11	0.0092	0.323
Fever (%)	34 (90)	46 (92)	1.35 (0.32–5.79)	0.17	0.0014	0.684
Anorexia (%)	28 (74)	48 (96)	8.57 (1.75–41.9)	9.51	0.079	0.008
Asthenia (%)	32 (84)	49 (98)	9.19 (1.06–79.93)	5.92	0.049	0.045
Pale skin (%)	9 (24)	11 (22)	0.91 (0.33–2.48)	0.03	0.0003	0.852
Weight loss (%)	37 (97)	50 (100)	NA	NA	NA	NA
Positive TST(%)	25 (66)	39 (78)	1.84 (0.72–4.75)	1.61	0.013	0.203
PTB-CXR (%)	35 (92)	32 (64)	0.15 (0.04–0.57)	10.38	0.086	0.005

*N* number of suspects in the group, *NA* not applicable

## Discussion

As in many developing countries, the diagnosis of PTB in the RoC relies on clinical symptoms examination, radiography and sputum smear microscopy. In a country with such a resource-limited health system, faster and more reliable methods such as automated liquid culture and nucleic acid amplification are not readily available. Also, in the RoC, where the burden of HIV and TB are high, SNPT cases are expected to be more frequent [21]. Patients diagnosed with SNPT are thought to be less infectious than those with SPPT; nevertheless, they are still able to transmit PTB [22]. The 2010 WHO guidelines recommend chest radiography (CXR) as the first step for cases suspected of TB having negative sputum smears. However, it ought to be noticed that diagnosis cannot solely rely on CXR as the quality of this exam is often compromised by poor film quality and variability in interpretation, particularly in HIV positive patients [23].

This study showed that pale skin, positive TST, weight loss and abnormal CXR were good indicators of PTB. Consistent with previous studies, 35 % of PTB patients were diagnosed with SNPT [24]. SNPT cases were more likely to show symptoms of anorexia and asthenia. A cough lasting more than 2 weeks was more prevalent in SPPT as seen in the literature [25]. On the opposite, a cough lasting less than 2 weeks could be symptomatic of SNPT. This led the WHO to change their recommendation for TB evaluation to include a cough of any duration replacing the previously conventional criteria of a persistent cough lasting more than 2 weeks [26]. Other clinical symptoms recommended by WHO include, hemoptysis (blood in sputum) and dyspnea (breathlessness)—and non-respiratory symptoms—such as chest pain, fever, chills, night sweats, anorexia, asthenia, and weight loss. These signs are somewhat ubiquitous in a clinical setting, thereby non-specific, and could possibly indicate a multitude of other illnesses which will confuse diagnosis of SNPT in countries such as RoC with limited options for fast, accurate and sensitive diagnostic methods.

We found that anorexia, asthenia and shorter duration cough were significantly identified in HIV infected patients with SNPT. In 2013, the WHO published guidelines for intensified case-finding in people living with HIV which took into account HIV status, AIDS severity and early CXR diagnosis in resource limited settings with high HIV/TB burden [26, 27]. Although CXR examination is an important component of SNPT diagnosis, it should be noted that its reliability can be affected by the HIV infection itself.

The results of this study hence confirm the need to propagate the use of improved point of diagnostics such as the Xpert MTB/RIF assay [28] which is based on nucleic acid amplification technique that detect the DNA of *Mycobacterium tuberculosis* and genetic mutations associated with resistance to the drug Rifampicin. From a single sputum sample, the result is obtained within 2 h. Since December 2010, WHO endorsed the Xpert MTB/RIF technology [29] with the expectation of earlier diagnosis and treatment initiation [30]. Unfortunately, only one Xpert MTB/RIF device is available in RoC [18]. In this country, only 2 % of the new TB cases were tested for multidrug resistance (MDR) and 17 % of tested patients were MDR-TB confirmed cases [18] which demonstrate the need for this technology. The national guidelines for Tuberculosis control in RoC recommend the use of Rifampicin and Isoniazid drugs (together with Ethambutol and Pyrazinamid) to treat new TB patients. Since Xpert MTB/RIF assay can test for resistance to Rifampicin, it should be routinely used in RoC to better diagnose TB and offer a better treatment to patients, especially for those having confirmed resistance to Rifampicin.

Recently, the use of the lateral flow urine TB lipoarabinomannan (LAM) antigen test (urine TB-LAM test for HIV infected patients was recommended in Uganda [31]. This rapid point-of-care test showed good performance in HIV-infected patients with advanced immune-suppression [32]. Such rapid diagnosis tools in HIV prevalent settings increases the ability to quickly identify patients who are at higher risk for death.

## Conclusion

A number of diagnosis tools can play an important role in finding SNPT cases. In a high TB and HIV prevalence setting, where culture and nucleic acid amplification technique are not routinely available, clinical signs and inexpensive examinations such as sputum smear microscopy, TST and chest X-ray radiography are of major interest in the diagnosis of PTB. However, it is necessary for the diagnosis to be done in a rigorous, quick and systematic manner since these signs are also indicative of a number of other diseases. Anorexia, asthenia and less persistent cough are good predictors of SNTP. These symptoms thus deserve to be recommended as indicators of SNPT diagnosis in country-level TB dispensaries in RoC. Morbidity and mortality associated with delayed diagnosis may be reduced with this strategy. These indicators are significantly of greater importance for HIV infected patients. In order to minimize over- and under-diagnosis of PTB, the use of diagnostic tools such as Xpert MTB/RIF assay and urine TB-LAM test should be expanded within the country.
